# Phononic Bandgap Optimization in Sandwich Panels Using Cellular Truss Cores

**DOI:** 10.3390/ma14185236

**Published:** 2021-09-11

**Authors:** Leonel Quinteros, Viviana Meruane, Eduardo Lenz Cardoso, Rafael O. Ruiz

**Affiliations:** 1 Department of Mechanical Engineering, Universidad de Chile, Av. Beauchef 851, Santiago 8370456, Chile; vmeruane@uchile.cl; 2 Millennium Nucleus on Smart Soft Mechanical Materials, Av. Beauchef 851, Santiago 8370456, Chile; 3 Department of Mechanical Engineering, Santa Catarina State University, R. Paulo Malschitzki 200, Joinville 89219710, Brazil; eduardo.cardoso@udesc.br; 4 Department of Civil Engineering, Universidad de Chile, Blanco Encalada 2002, Santiago 8370449, Chile; rafaelruiz@uchile.cl; 5 Uncertainty Quantification Group, Center for Modern Computational Engineering, Universidad de Chile, Blanco Encalada 2002, Santiago 8370449, Chile

**Keywords:** sandwich panel, size optimization, smart-material, phononic structure

## Abstract

The development of custom cellular materials has been driven by recent advances in additive manufacturing and structural topological optimization. These contemporary materials with complex topologies have better structural efficiency than traditional materials. Particularly, truss-like cellular structures exhibit considerable potential for application in lightweight structures owing to their excellent strength-to-mass ratio. Along with being light, these materials can exhibit unprecedented vibration properties, such as the phononic bandgap, which prohibits the propagation of mechanical waves over certain frequency ranges. Consequently, they have been extensively investigated over the last few years, being the cores for sandwich panels among the most important potential applications of lattice-based cellular structures. This study aims to develop a methodology for optimizing the topology of sandwich panels using cellular truss cores for bandgap maximization. In particular, a methodology is developed for designing lightweight composite panels with vibration absorption properties, which would bring significant benefits in applications such as satellites, spacecraft, aircraft, ships, automobiles, etc. The phononic bandgap of a periodic sandwich structure with a square core topology is maximized by varying the material and the geometrical properties of the core under different configurations. The proposed optimization methodology considers smooth approximations of the objective function to avoid non-differentiability problems and implements an optimization approach based on the globally convergent method of moving asymptotes. The results show that it is feasible to design a sandwich panel using a cellular core with large phononic bandgaps.

## 1. Introduction

Various natural materials exhibit properties that cannot be realized in conventional materials employed in structural engineering. This is the result of the evolution of these natural materials over millions of years, resulting in an optimized architecture. As an example, the internal architecture of bones provides them with exceptional structural efficiency that has not be replicated with artificial materials.

Despite the fact that the concept of elastic tailoring of materials is not new, the fabrication of cellular materials with increasingly complex topologies has been driven by the development of additive manufacturing technologies [1]. Moreover, the application of topological optimization enabled the design of material with desired properties such as reduced weight and high strength or stiffness. In this context, lattice-based materials have proven to reach an exemplary relationship among stiffness, strength, and density [2], which makes them ideal for low-weight design applications (e.g., for aircraft applications [3]). The architecture of lattice materials is composed by distinct structural elements, including slender beams and struts, which are arranged in characteristic unit cells periodically distributed in space.

Among the potential applications of lattice-based cellular materials, cores for sandwich panels are included [1]. Sandwich panels are composed of a lightweight core sandwiched between two skins, resulting in a stiff structure at a reduced weight. Therefore, sandwich structures are frequently used in applications where lightweight materials are crucial, such as satellites, spacecraft, aircraft, automobiles, ships, rail cars, wind energy systems, and bridges [4].

Additive manufacturing techniques offer the possibility of manufacturing tailored cores for sandwich structures with superior properties than conventional foam or honeycomb cores. For example, it widens the range of possible core architectures; in addition, the cores can be designed to achieve multiple objectives (such as mechanical, thermal, or wave propagation, among others). In addition to their attractive static properties, architected cellular materials can also be design to suppress the propagation of mechanical waves, which may be achieved via phononic bandgaps [5]. Phononic structures can be used to reduce mechanical wave propagation in a material over a certain frequency range. As a result, these structures have been extensively investigated over the last few years [6].

Jensen [7] was among the first to demonstrate that truss-like periodic structures exhibit bandgaps, which suppress the propagation of elastic waves at certain frequencies. He also showed that by combining periodic and homogeneous structures, wave guides may be obtained. Ruzzene and Scarpa [8] studied the presence of bandgaps in auxetic and honeycomb lattices, which are composed by an array of linked struts. They found that the internal angle of the auxetic and honeycomb lattices significantly affects bandgaps. Liebold-Ribeiro and Körner [5] investigated the bandgap behaviors of bidimensional cellular structures with hexagonal, quadratic and chiral lattice types, concluding that these materials may exhibit considerable bandgaps. Warmuth and Körner [9] reached a similar conclusion and reported that by replacing straight struts with bent ones (chiral), bandgaps are achieved. Wang et al. [10] studied the dependence of bandgap width and frequency with the lattice topology. Characterizing the topology by the number of connections at the joints, known as the lattice coordination number. They concluded that large coordination numbers facilitate the formation of bandgaps because, in these cases, the struts act as mechanical resonators. The bandgap width decreases with the coordination number until a certain threshold from which no bandgap is generated.

The mentioned investigations show that by modifying the material or geometrical properties of the struts, the corresponding bandgaps of a lattice material can be manipulated. Accordingly, it becomes reasonable to implement topology optimization techniques to design lattice structures to meet desired bandgap properties. Several studies have investigated the use of topology optimization algorithms to obtain optimal configurations that yield the largest bandgap. However, these studies have focused mainly on the design of two-dimensional structures [11,12].

During topology optimization, the geometrical and/or material properties of each element in the design domain are designated as design variables. The optimization then finds the microstructure that maximizes the bandgap (distance) between two bands in the phononic band diagram. The first application of topology optimization for bandgap maximization was presented by Sigmund and Jensen [13], designing a two-dimensional phononic solid structure constructed by two materials, wherein the design parameters were the relative density of each element in the structure. Gazonas et al. [14] used genetic algorithms (GAs) as an optimization tool to determine the optimal distribution of a two-dimensional two-phase structure. In this case, the optimization variables are binary numbers, representing the material property as zero-one variables. Hussein et al. [15] used a similar approach to maximize the sum of the bandgap widths in a two-dimensional periodic bimaterial. Liu et al. [16] implemented a two-stage methodology using GAs to maximize the bandgap of a two-dimensional solid structure. In the first step, the unit cell was divided into a 20 by 20 pixel grid, the solution of the first stage was used as the initial searching point for the second stage, with 40 by 40 pixels. Bilal and Hussein [17,18] presented a specialized GA to design two-dimensional phononic materials with in-plane and out-of-plane waves. Dong et al. [19] showed that wider bandgaps are obtained in two-dimensional phononic materials when no assumption is made regarding the cell symmetry compared to the symmetrical case. Diaz et al. [20] studied the presence of bandgaps in a two-dimensional grid structure consisting of intersecting struts. They maximized the width of the bandgap by adding nonstructural masses at key grid positions while leaving the grid shape unaltered. Halkjær et al. [21] applied topology optimization to maximize the bandgaps in bi-material beams and plates. In a later work, they maximized the bandgap in plates with one material [22]. The optimal design was adopted to manufacture a polycarbonate plate built with 10 by 10 unit cells; the experimental frequency response function (FRF) showed a clear bandgap. Vanatabe et al. [23,24] maximized the bandgap of a functionally graded two-dimensional piezocomposite periodic material through topology optimization. Their results show that the resulting solutions can be post-processed to obtain zero-one values without affecting significantly the band diagram. Li et al. [25] proposed a new optimization approach based a bidirectional evolutionary structural optimization (BESO) for bandgap maximization of two-dimensional structures. A gradient-based optimization algorithm was used by Yi et al. [26] to maximize the bandgap at a given frequency for two-dimensional solid materials. This approach makes it possible to design a bandgap with the maximum width for a target frequency.

Some investigations have implemented multi-objective optimization strategies for phononic materials with mass or stiffness restrictions. Dong et al. [27] implemented a multi-objective optimization based on GAs to maximize the bandgap width of two-dimensional solid phononic materials with minimum mass. A similar multi-objective GA optimization strategy was used by Hedayatrasa et al. [28] to maximize the bandgap width and in-plane stiffness of perforated solid plates. Hedayatrasa et al. [29] maximized the bandgap width in conjunction with the bandgap gradient induced by deformation using multi-objective GA optimization. This implementation allowed them to tune the bandgap under equibiaxial stretching. Li et al. [30] implemented a simultaneous maximization of the bandgap width and the shear modulus of two-dimensional solid materials using the BESO algorithm.

Evolutionary optimization algorithms such as GAs are more eficient for problems with few design variables. As the computational cost of these algorithms largely depends on the number of design variables, the objective function must be evaluated several times. Therefore, in problems with a large amount of design variables, gradient-based optimization approaches are desirable, even with the possibility of obtaining local optima [11]. Gradient-based optimization approaches for band-gap maximization require the computation of eigenvalue sensitivities, which becomes a problem in the presence of repeated eigenvalues, as they are not differentiable. To handles this, Torii and De Faria [31] proposed the P-norm as a smooth approximation of the objective function, which also solves the mode switching problem. This formulation was applied by Quinteros et al. [32] to maximize the bandgap of a two-dimensional truss-like cellular structure, thereby demonstrating its effectiveness in avoiding convergence problems.

Sandwich panels with lattice-based cellular cores are extremely desirable for applications that require multi-functional materials, such as lightweight ultra-stiff structural materials. The optimal cell that maximizes the stiffness/weight ratio in these type of panels has been investigated by [33,34]. However, to the author’s knowledge, the design of such panels with vibration attenuation properties (i.e., a bandgap) has not yet been explored. Research on phononic crystals has focused mainly on two-dimensional materials, and sandwich panels with vibration absorption properties have been obtained mainly by using internal resonators (i.e., internally resonant metastructures) [35,36].

The aim of this investigation is to develop an efficient methodology for designing ultralight sandwich panels with cellular truss cores and large phononic bandgaps. The phononic bandgap of a periodic sandwich structure with a regular core topology was maximized by varying both the material and the geometrical properties under different core configurations. The proposed optimization methodology considers smooth approximations of the objective function to avoid non-differentiability problems and uses the globally convergent method of moving asymptotes (GCMMA) optimization algorithm. The remainder of this article is organized as follows. Section 2 introduces the theory needed to understand the periodic structure to be investigated. Section 3 shows the geometry that will be optimized and all assumptions related to it. Section 4 states the optimization problem required to maximize bandgaps. Section 5 provides the results obtained using this approach, and Section 6 provides concluding remarks.

## 2. Periodic Structures Theory

**Figure 1:**
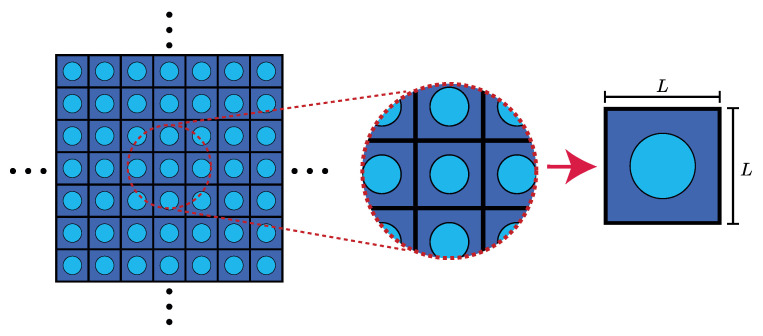
Periodic structure and a repeated square pattern with edge length *L*.

To understand periodic structure theory (PST), it is necessary to establish the equations used to describe the phenomenon of mechanical wave propagation in periodic materials. Let us consider a continuum two-dimensional (2D) structure, as shown in Figure 1. In this example, a square cell with edge length *L* has been chosen to tile an infinite 2D space. The structure does not necessarily need to be a square, and it could possess a complex 2D [37] or 3D [38] geometry.

**Figure 2:**
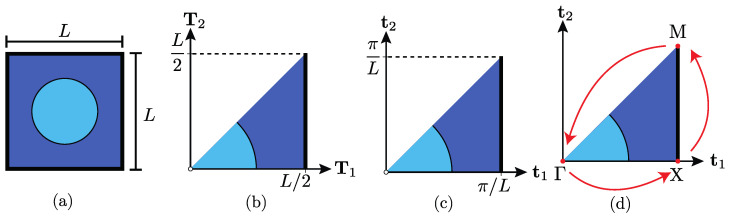
Periodic cell and IBZ. (**a**) Periodic cell. (**b**) Direct Lattice. (**c**) Reciprocal lattice. (**d**) IBZ.

The periodic square geometry extracted from Figure 1. is studied in Figure 2a. Three reflexive symmetries can be depicted: horizontal, vertical, and 45° symmetries. Considering these symmetries and locating the reference system at the center of the structure, this structure can be represented using a reduced zone, as illustrated in Figure 2b. An important concept in PST corresponds to the definition of a reciprocal space, which is described by the vectors $t_{1}$ and $t_{2}$ considering the structure length as 2π/L (for further information see ref. [39]). Figure 2c shows the same information as Figure 2b, but in the reciprocal space.

The last important concept is the wave vector k, which describes the wavelength and direction of wave propagation. This wave vector is evaluated at the path that follows the perimeter defined by the corners of the triangle in the reciprocal space. This path begins at Γ, passes through *X* and *M*, and returns to Γ, as presented in Figure 2d. It can be written as

(1) $$k=k_{x}\hat{i}+k_{y}\hat{j},$$

for 2D problems.

The path conducted by the vector is also referred to as the irreducible Brillouin zone (IBZ) [40]. According to the Floquet–Bloch wave theory, the displacement in the reciprocal space for a periodic unit cell is given by

(2) $$u\left(X+r\right)=u\left(X\right)e^{ikr},$$

where r is the cell periodicity, i the imaginary unit, u is the displacement, and k is the wave vector. Equation (2) will be used to impose the periodic boundary conditions in the finite element model of the sandwich panel, which will be explained in subsequent sections of this article.

## 3. Sandwich Panel Modelling

### 3.1. Geometry

**Figure 3:**
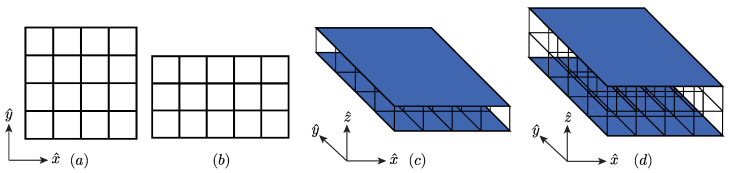
Periodic structure and the repeating pattern, (**a**) $n_{x}=n_{y}=4$ and $n_{z}=0$, (**b**) $n_{x}=5$, $n_{y}=3$, and $n_{z}=0$, (**c**) $n_{x}=n_{y}=4$ and $n_{z}=1$, (**d**) $n_{x}=n_{y}=4$ and $n_{z}=2$.

There are numerous methods of geometrically arranging the core of the sandwich panel. In this research, a rectangular lattice structure will be employed as illustrated in Figure 3. The topology of the core is described by the number of cells in the directions $\hat{x}$, $\hat{y}$ and $\hat{z}$ using the variables $n_{x}$, $n_{y}$, $n_{z}$, respectively. Furthermore, the length in each direction is denoted with $L_{x}$, $L_{y}$, and $L_{z}$. A scheme of this configuration is presented in Figure 3. Here, Figure 3a,b show the in-plane unit cells (considering $n_{z}=0$) using $n_{x}=n_{y}=4$ in the first case and $n_{x}=5$ and $n_{y}=3$ in the second. The sandwich panel unit cells with $n_{z}>0$ are illustrated in Figure 3c,d, in particular for $n_{x}=n_{y}=4$. Note that Figure 3c,d use $n_{z}=1$ and $n_{z}=2$, respectively. Therefore, these three parameters are used to control the number of cells, specifically to allow a wider range of possible geometries. The cross-section of the struts will be supposed to be circular with a diameter *D*.

### 3.2. Finite Element Model

**Figure 4:**
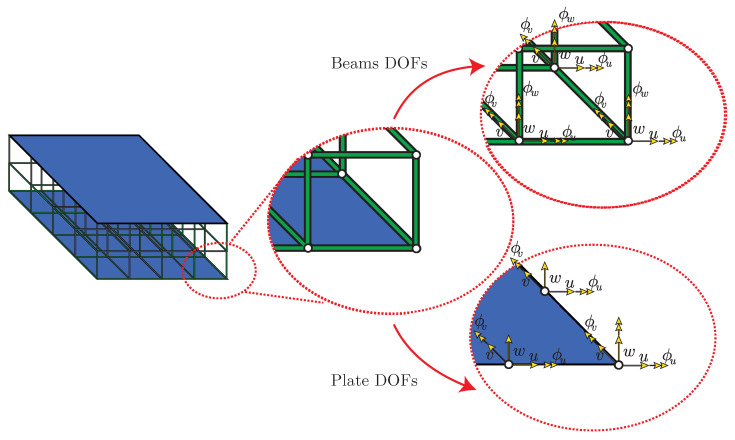
Sandwich plate surfaces showing the DOFs of the nodes on the beams and plate elements.

The structural model is built by adopting a finite element approach. The skins are modeled using four-node Reissner–Mindlin elements with five degrees of freedom (DOF) per node, which consist of three displacements in the axis directions *u*, *v*, and *w*, and two rotations $\phi_{u}$ and $\phi_{v}$ [41]. The uni-dimensional elements are modeled using 3D Timoshenko beam elements, with two nodes per element and six degrees of freedom per node (three rotations and three displacements), as shown in Figure 4.

**Figure 5:**
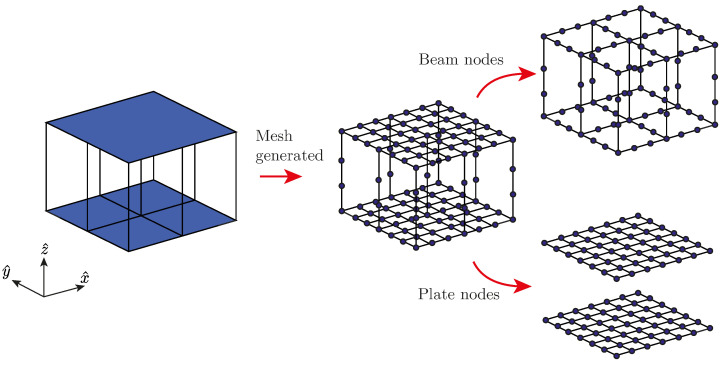
Mesh generated for a $n_{x}=n_{y}=2$ and $n_{z}=1$ sandwich panel using a refinement parameter η=3.

The accuracy of the model is evaluated through a verification process based on the mesh refinement. Owing to geometrical considerations, this refinement will be achieved using a variable denoted as η, which will divide each bar into η sections, as shown in Figure 5 for a core with $n_{x}=n_{y}=2$, $n_{z}=1$, and η=3. Material properties and diameter *D* of a given bar *r* are then associated to the design variables $x_{r}^{M}$ and $x_{r}^{A}$, respectively, for any value of η. The importance of this refinement will be discussed in the results section using a mesh dependency analysis.

### 3.3. PST Applied to the Sandwich Panel

**Figure 6:**
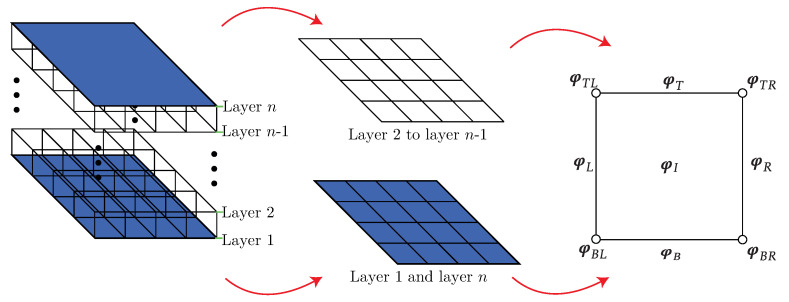
Regions considered to impose periodicity in the unit cell.

The PST is implemented to infinitely replicate the sandwich panel unit cells shown in Figure 3 in the $\hat{x}$-$\hat{y}$ plane. Taking into consideration Figure 6, the nodes in each layer are split to implement the Floquet–Bloch periodic condition. The nodes are split into sets $\varphi_{L}$, $\varphi_{R}$, $\varphi_{B}$, $\varphi_{T}$, and $\varphi_{I}$, which are the left, right, bottom, top edges, and interior nodes, respectively. Depending on the layer, these nodes may be in the beam elements or in both the plate and beam elements, as shown in Figure 6.

According to the methodology proposed by Langlet et al. [42], the infinite periodicity of the unit cell is established by the boundary conditions presented in Equation (2). As was defined in Section 2, the wave vector for a two-dimensional problem is expressed as Equation (1), which is a vector that runs along the perimeter of the IBZ. The Floquet–Bloch condition is applied to both the displacement and rotational degrees of freedom. Therefore, for each layer *j*-th, the boundary conditions are defined as

(3) $$\begin{array}{cc} \; & \varphi_{T}^{j}=\varphi_{B}^{j}e^{iLk_{y}}\\ \; & \varphi_{R}^{j}=\varphi_{L}^{j}e^{iLk_{x}}\\ \; & \varphi_{BR}^{j}=\varphi_{BL}^{j}e^{iLk_{x}}\\ \; & \varphi_{TL}^{j}=\varphi_{BL}^{j}e^{iLk_{y}}\\ \; & \varphi_{TR}^{j}=\varphi_{BL}^{j}e^{iL(k_{x}+k_{y})} \end{array},$$

where the superscript represents the *j*-th layer and φ is a vector of the rotational or displacement degrees of freedom. The subscript *L* represents all the nodes in the left edge, *R* is used for the right edge nodes, *B* for the bottom edge nodes, and *T* for the top edge nodes. The degrees of freedom over the corners are denoted by the subscripts BL, BR, TL, and TR according to Figure 6. All of the internal degrees of freedom in the *j* layer are arranged in the vector $\varphi_{I}^{j}$.

Owing to the Bloch relation, the number of independent degrees of freedom can be reduced by using the relationship $\varphi^{j}=T^{j}\tilde{\varphi^{j}}$, where $\varphi^{j}$ is the vector of all nodal degrees of freedom, $\tilde{\varphi }$ is the reduced vector, and $T^{j}$ is a matrix mapping both vectors. Vectors $\varphi^{j}$ and $\tilde{\varphi^{j}}$ are expressed as

(4) $$\varphi^{j}=\left[\begin{array}{c} \varphi_{L}^{j}\\ \varphi_{R}^{j}\\ \varphi_{B}^{j}\\ \varphi_{T}^{j}\\ \varphi_{BL}^{j}\\ \varphi_{TL}^{j}\\ \varphi_{BR}^{j}\\ \varphi_{TR}^{j}\\ \varphi_{I}^{j} \end{array}\right];\;\;\;{\tilde{\varphi }}^{j}=\left[\begin{array}{c} \varphi_{L}^{j}\\ \varphi_{B}^{j}\\ \varphi_{BL}^{j}\\ \varphi_{I}^{j} \end{array}\right],$$

while the matrix T for the *j*-th layer is given by

(5) $$\begin{array}{c} T^{j}=\left[\begin{array}{cccc} I & 0 & 0 & 0\\ Ie^{iLk_{x}} & 0 & 0 & 0\\ 0 & I & 0 & 0\\ 0 & Ie^{iLk_{y}} & 0 & 0\\ 0 & 0 & I & 0\\ 0 & 0 & Ie^{iLk_{y}} & 0\\ 0 & 0 & Ie^{iLk_{x}} & 0\\ 0 & 0 & Ie^{iL\left(k_{y}+k_{x}\right)} & 0\\ 0 & 0 & 0 & I \end{array}\right], \end{array}$$

where I is the identity matrix.

Considering the assemblage of the entire sandwich cell structure using the finite element method, the matrix T can be written as

(6) $$T=\left[\begin{array}{ccccccc} T^{1} & 0 & 0 & \cdots & 0 & 0 & 0\\ 0 & T^{2} & 0 & \cdots & 0 & 0 & 0\\ 0 & 0 & T^{3} & \cdots & 0 & 0 & 0\\ \vdots & \; & & \ddots & \; & & \vdots \\ 0 & 0 & 0 & \cdots & T^{n-2} & 0 & 0\\ 0 & 0 & 0 & \cdots & 0 & T^{n-1} & 0\\ 0 & 0 & 0 & \cdots & 0 & 0 & T^{n} \end{array}\right].$$

Lastly, the classical eigenvalue problem taking periodicity into account is expressed as

(7) $$\left(T^{T}KT-\omega_{i}^{2}T^{T}MT\right)u_{i}=\left(K^{\prime }-\omega_{i}^{2}M^{\prime }\right)u_{i}=0,$$

where K and M represent the global stiffness and mass matrices, respectively, $\omega_{i}^{2}$ is the *i*th eigenvalue ($\lambda_{i}=\omega_{i}^{2}$), and $u_{i}$ corresponds to the *i*th eigenvector related to the corresponding wave vector.

The dependence to the wave vector k is introduced to the problem by the mapping matrix T. Therefore, the eigenvalues can be computed for different values of the wave vector in the path Γ−M−X−Γ to construct the band diagram. This diagram provides a characterization of the dynamic response of the periodic structure as a function of k, visualizing which wavelengths can be propagated along an infinite arrangement of cells.

**Figure 7:**
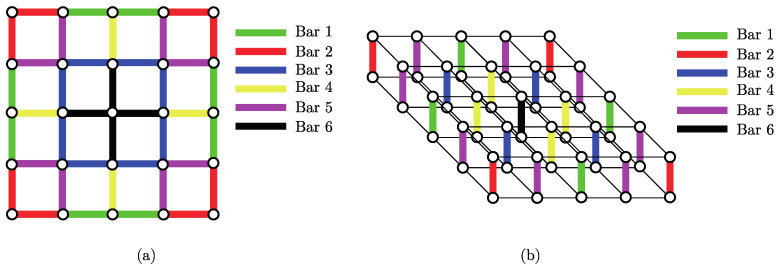
Regions considered to impose periodicity of the periodic unit cell for (**a**) in plane bars and perpendicular bars (**b**).

Finally, using the symmetries shown in Figure 2, it is possible to diminish the number of optimization variables related to the bars by forcing the unit cell to accomplish the symmetries, as illustrated in Figure 7. Note that even though the colors are the same in Figure 7, the bars in both subfigures are independent.

## 4. Optimization Problem

### 4.1. Design Variables

During the optimization process that will be defined, only the material and beam cross-sections are selected as design variables, leaving the plate elements aside. Each parameter is described by an interpolation function between two possible values. The selection of this function is not trivial and must be undertaken carefully, because it affects the physics of the problem [43]. However, for band gap optimization the linear function is sufficient, as the solution tends to take values at the limits of the ranges [44]. Thus, the parametrizations for the material properties, density (ρ), elastic modulus (*E*) and Poisson’s ratio (ν) are

(8) $$\rho =\left(\rho_{2}-\rho_{1}\right)x_{r}^{M}+\rho_{1},$$

(9) $$E=\left(E_{2}-E_{1}\right)x_{r}^{M}+E_{1},$$

and

(10) $$\nu =\left(\nu_{2}-\nu_{1}\right)x_{r}^{M}+\nu_{1},$$

where the $x_{r}^{M}\in \left[0,1\right]$ controls the material properties for each bar *r*. For the area properties, a circular cross-section with diameter *D* is used,

(11) $$D=\left(D_{2}-D_{1}\right)x_{r}^{A}+D_{1}$$

where $x_{r}^{A}\in \left[0,1\right]$ and controls the cross-section properties. This variable modifies properties such as the inertia moments $I_{x}$, $I_{y}$, and $I_{z}$, and the area *A*.

### 4.2. Optimization

**Figure 8:**
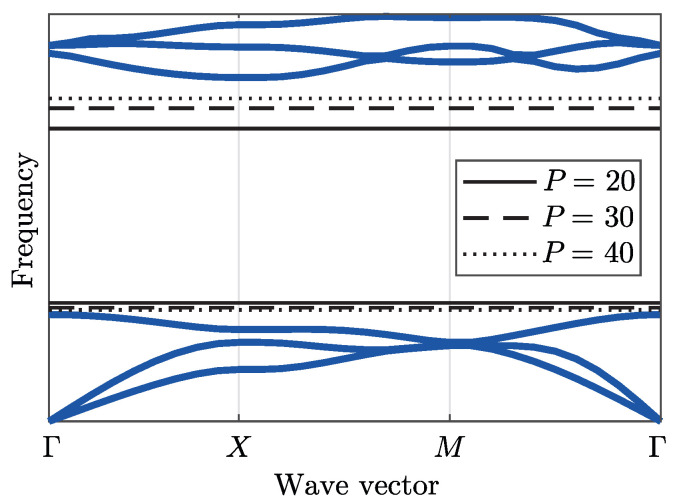
Example of P-norm smooth approximations of the minimum and maximum values of the frequency for n=3 and m=6.

Given all of the background information necessary to understand the band diagram (also referred to as a dispersion diagram), the optimization process may now be considered. The objective is to determine the unit cell that maximizes the bandgap, which is calculated by the distance between two consecutive bands *n* and n+1. Figure 8 illustrates an example of a band diagram, which possesses six bands (blue lines) and a gap between the third and fourth bands, referred as the bandgap. Therefore, the bandgap width to be maximized is the distance between the band minimum $\omega_{n+1}\left(x,k\right)$ and the band maximum $\omega_{n}\left(x,k\right)$ for k∈[Γ,X,M,Γ]. The design variable vector x accounts for both $x^{D}$ and $x^{M}$, such that

(12) $$x=\left\{\begin{array}{c} x^{D}\\ x^{M} \end{array}\right\};$$

the first term is related to the bars area $x^{D}$ and the second defines the bars material $x^{M}$. The wave vector k is defined along the perimeter of the IBZ. The resulting formulation is expressed as

(13a) $$\begin{array}{cccc} & \;\max_{x} & \; & \frac{\min\omega_{n+1}\left(x,k\right)-\max\omega_{n}\left(x,k\right)}{\bar{\omega }} \end{array}$$

(13b) $$\begin{array}{cccc} & \mathrm{subject}\;\mathrm{to} & & (K^{\prime }\left(k\right)-\omega^{2}M^{\prime }\left(k\right))u=0 \end{array}$$

(13c) $$\begin{array}{cccc} & & & k\in \;IBZ \end{array}$$

(13d) $$\begin{array}{cccc} & & & 0\leq x_{i}\leq 1 \end{array}$$

where the objective function is scaled by the mean frequency in which the bandgap is tuned, defined as

(14) $$\bar{\omega }=\frac{\min\omega_{n+1}\left(x,k\right)+\max\omega_{n}\left(x,k\right)}{2}.$$

Additionally, it should be noted that the normalized objective function is an indicator of how the bandgap improves relative to the mean bandgap frequency because not only is the bandgap width important, but it is also crucial to know where it is located.

An issue that may occur in eigenvalue optimization problems is the non-differentiability related to repeated eigenvalues [45]. Other problem that occur when targeting individual eigenvalues is mode switch during optimization. To overcome these issues, Quinteros et al. [32] proposed the use of P-norms as smooth approximations for both $\max\omega_{n}$ and $\min\omega_{n+1}$ in the form

(15) $$\max\omega_{n}\left(x,k\right)\approx {∥\omega_{n}\left(x\right)∥}_{P}={\left(\sum_{j=1}^{n}\sum_{i=1}^{PIBZ}\omega_{ij}^{P}\left(x\right)\right)}^{1/P},$$

and

(16) $$\min\omega_{n+1}\left(x,k\right)\approx {∥\omega_{n+1}\left(x\right)∥}_{-P}={\left(\sum_{j=n+1}^{m}\sum_{i=1}^{PIBZ}\omega_{ij}^{-P}\left(x\right)\right)}^{-1/P}.$$

In both expressions, the first sum to PIBZ is associated with the discretization of the wave vector k, while the index *j* refers to the *j*-band.

In Equation (15), the sum that ranges from j=1 to *n* represents the maximum value for bands 1 to *n*. This indicates that all the eigenvalues in this range are considered. On the other hand, Equation (16) represents the minimum value for bands n+1 to *m*. In both approximations a finite set of eigenvalues are combined, thereby hindering mode switching inside of each set. Moreover, it is possible there is mode switching occurring between both sets during optimization. This approach also resolves the non-differentiability of repeated eigenvalues, as discussed in [31]. The optimization problem may now be stated as

(17a) $$\begin{array}{cccc} & \;\max_{x} & \; & 2\frac{{∥\omega_{n+1}\left(x\right)∥}_{-P}-{∥\omega_{n}\left(x\right)∥}_{P}}{{∥\omega_{n+1}\left(x\right)∥}_{-P}+{∥\omega_{n}\left(x\right)∥}_{P}} \end{array}$$

(17b) $$\begin{array}{cccc} & \mathrm{subject}\;\mathrm{to} & & \left(K^{\prime }(k\right)-\omega^{2}M^{\prime }\left(k\right))u=0\;\;\;. \end{array}$$

(17c) $$\begin{array}{cccc} & & & k\in \;IBZ \end{array}$$

(17d) $$\begin{array}{cccc} & & & 0\leq x_{i}\leq 1 \end{array}$$

Figure 8 illustrates a band diagram with the presence of a bandgap. Equations (15) and (16) are evaluated for different values of *P*, considering n=3 and m=6. It is observed that as *P* increases, Equation (16) converges to the true minimum and Equation (15) to the true maximum despite intersections (repeated values) in the band diagram.

### 4.3. Sensitivity Analysis

In this study, a gradient-based solver is used to optimize the bandgap. The derivatives of Equations (15) and (16) with respect to a design variable $x_{r}^{\alpha }$ are given by

(18) $$\frac{d{∥\omega_{n}∥}_{P}}{dx_{r}^{\alpha }}={\left(\sum_{j=1}^{n}\sum_{i=1}^{PIBZ}\omega_{ij}^{P}\right)}^{\frac{1-P}{P}}\left(\sum_{j=1}^{n}\sum_{i=1}^{PIBZ}\omega_{ij}^{P-1}\frac{d\omega_{ij}}{dx_{r}^{\alpha }}\right)$$

and

(19) $$\frac{d{∥\omega_{n+1}∥}_{-P}}{dx_{r}^{\alpha }}={\left(\sum_{j=n+1}^{m}\sum_{i=1}^{PIBZ}\omega_{ij}^{-P}\right)}^{\frac{-(1+P)}{P}}\left(\sum_{j=n+1}^{m}\sum_{i=1}^{PIBZ}\omega_{ij}^{-P-1}\frac{d\omega_{ij}}{dx_{r}^{\alpha }}\right),$$

where α could be *D* (geometry) or *M* (material). The eigenvalue sensitivities are given by [46],

(20) $$\frac{d\omega_{ij}}{dx_{r}^{\alpha }}=\frac{u_{ij}^{T}\left(\frac{dK^{\prime }}{dx_{r}^{\alpha }}-\omega_{ij}^{2}\frac{dM^{\prime }}{dx_{r}^{\alpha }}\right)u_{ij}}{2\omega_{ij}u_{ij}^{T}Mu_{ij}}.$$

Here, the matrices $K^{\prime }$ and $M^{\prime }$ represent the stiffness and mass matrices of the entire structure, respectively. Derivation with respect to $x_{r}^{\alpha }$ must be performed on each matrix component. However, these elements are independent, and if the stiffness and mass matrices of the element *e* are denoted using $K_{e}$ and $M_{e}$, respectively, the derivatives with respect the element *r* are given by

(21) $$\frac{dK_{e}}{dx_{r}^{\alpha }}=\delta_{er}\frac{dK_{e}}{dx_{r}^{\alpha }},$$

and

(22) $$\frac{dM_{e}}{dx_{r}^{\alpha }}=\delta_{er}\frac{dM_{e}}{dx_{r}^{\alpha }},$$

where $\delta_{er}$ denotes the Kronecker’s delta. Finally, the sensitivity of the objective function can be calculated as

(23) $$\frac{d}{dx_{r}^{\alpha }}\left(f_{obj}\left(x\right)\right)=\frac{d}{dx_{r}^{\alpha }}\left(2\frac{{∥\omega_{n+1}∥}_{-P}-{∥\omega_{n}∥}_{P}}{{∥\omega_{n+1}∥}_{-P}+{∥\omega_{n}∥}_{P}}\right)=4\frac{\left(\frac{d{∥\omega_{n+1}∥}_{-P}}{dx_{r}^{\alpha }}{∥\omega_{n}∥}_{P}-\frac{d{∥\omega_{n}∥}_{P}}{dx_{r}^{\alpha }}{∥\omega_{n+1}∥}_{-P}\right)}{{\left({∥\omega_{n+1}∥}_{-P}+{∥\omega_{n}∥}_{P}\right)}^{2}}.$$

## 5. Results

In this section, the optimization parameters and results are provided. The material and geometrical considerations are presented in Table 1, in which aluminum and tungsten are used for the core and aluminum sheets are used in the sandwich skins. Those materials were used owing to the high contrast between their properties. The solver selected for the optimization is the GCMMA [47] and its parameters are the ones used in ref. [48]. The number of external iterations of the GCMMA is fixed to 100. The initial seed was selected using the Latin hypercube sampling method [49] to evenly map the design space, as this problem is non-convex and may converge to a local minimum. For the following study cases, 100 Latin hypercube initial points were used, and the best result among those is reported on each case.

### 5.1. Convergence: Results Verification

**Figure 9:**
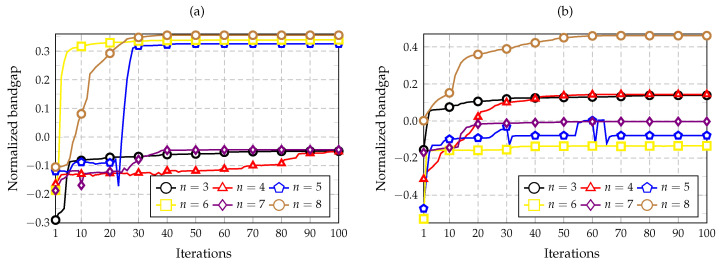
Iteration history obtained for n=3 to 8. (**a**) $n_{c}=2$ and $n_{z}=1$. (**b**) $n_{c}=2$ and $n_{z}=2$. Bands from n=3 to 8 are optimized in each case.

**Figure 10:**
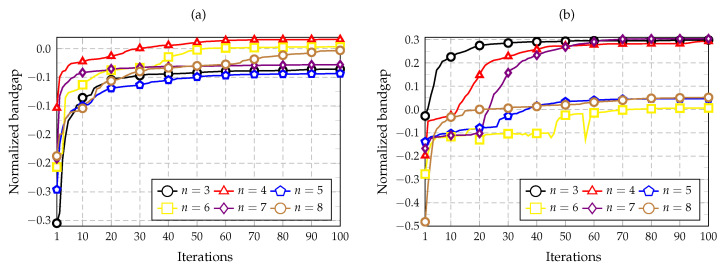
Iteration history obtained for n=3 to 8. (**a**) $n_{c}=4$ and $n_{z}=1$. (**b**) $n_{c}=4$ and $n_{z}=2$. Bands from n=3 to 8 are optimized in each case.

**Figure 11:**
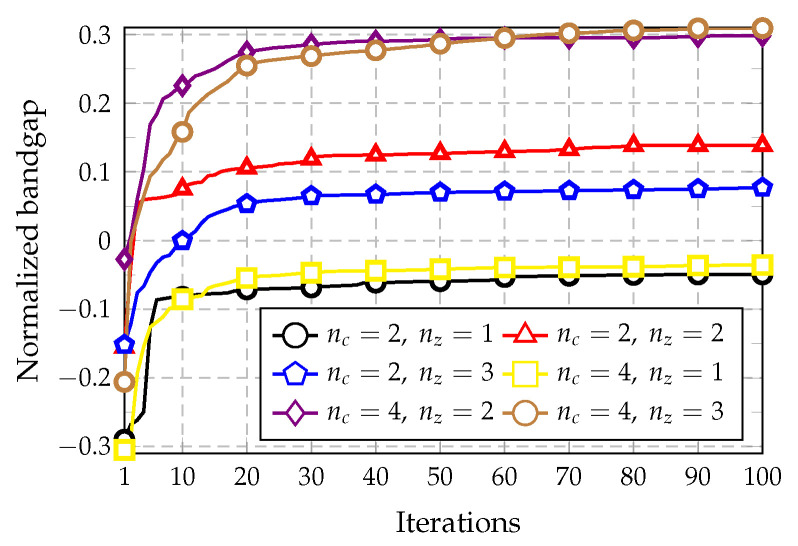
Iteration history obtained for $n_{c}=2$ and 4 with $n_{z}=1,2,$ and 3. In this case, only the band n=3 is optimized.

The convergence of the optimization algorithm is investigated by using different combinations of $n_{c}$ and $n_{z}$, wherein the objective is to maximize the bandgap above bands 3 to 8 (n=3 to n=8). The convergence plots are shown in Figure 9, Figure 10 and Figure 11, in which the value of the objective function is plotted along the iterations of the optimization process.

Figure 9 presents the convergence plots for the combinations $n_{c}=2$ and $n_{z}=1$ or 2, whereas Figure 10 shows the iteration results for the combinations $n_{c}=4$ and $n_{z}=1$ or 2. Lastly, Figure 11 compares the convergence plots for $n_{c}=2$ and 4 considering $n_{z}=1$ to 3, using the same band for optimization (n=3). The last case is performed to study how the complexity of the cell influences the optimization. It should be noted that negative values of the normalized bandgap indicate that no bandgap exists because the bands are overlapping.

The plots presented in Figure 9a show that the best bands are n=5, 6, and 8 for this optimization goal, in which the convergence was obtained in less than 50 iterations. The three curves achieve similar normalized bandgap values of approximately 0.33 and 0.36. In Figure 9b, the best results were obtained for n=8, followed by those for n=3 and n=4. The value achieved for n=8 was the maximum among all the other convergence plots, with a normalized bandgap of 0.46. Considering Figure 10a, only one positive normalized bandgap value was obtained for n=4 with a value of 0.016. By analyzing Figure 10b, the three bands of n=3, 4, and 7 show similar results with an optimum value near 0.3. Finally, considering the plot shown in Figure 11, the best maximum values are achieved by using $n_{c}=4$ and considering $n_{z}=2$ and 3.

A poor refinement η=3 was employed during the optimization process to minimize computational costs. Because beam and plate elements are selected to model the sandwich plate behavior, the mesh sensitivity must be carefully studied. Additionally, a post-process must be performed to obtain values on the limits of the design space. This is required because even when the optimum value tends towards the extreme values of the interpolation presented in Equations (8)–(11), the material properties must be strictly 0 or 1 (if the material variables do not accomplish this, the material is not physically possible). Therefore, the design variables related to the material properties will be approximated to the nearest value of 1 or 0. However, the area properties may violate the 1 or 0 criterion post-processing considering that these variables are not restricted in the same manner as the material properties. Therefore, the post-processing will be applied only to the material properties.

Table 2 and Table 3 show how the refinement and post-processing influence the bandgap for the best results obtained using different combinations of $n_{z}$ and $n_{c}$, which were determined previously. The P-norm columns presented in Table 2 and Table 3 show the final values obtained by the optimization process after 100 iterations. The material properties of that solution vector are then post-processed and the bars are refined as described in Section 3.2. It is worth noting that the best P-norm value does not imply that the configuration will have the best post-processed value; this is due to the poor refinement. Even when that refinement does not provide the best approach for obtaining a reliable value, it estimates good candidates with relatively low computational costs. However, there are some configurations in which the bandgap does not proliferate after refinement. For example, when the p-norm value obtained with $n_{c}=2$, $n_{z}=2$, and n=8 is refined, the bandgap is lost completely. Nevertheless, all of the normalized bandgap configurations tend to converge.

The best bandgaps were obtained with the configurations $n_{c}=2-n_{z}=1$ and $n_{c}=4-n_{z}=2,3$.

### 5.2. Band Diagram and Lattice Topology

**Figure 12:**
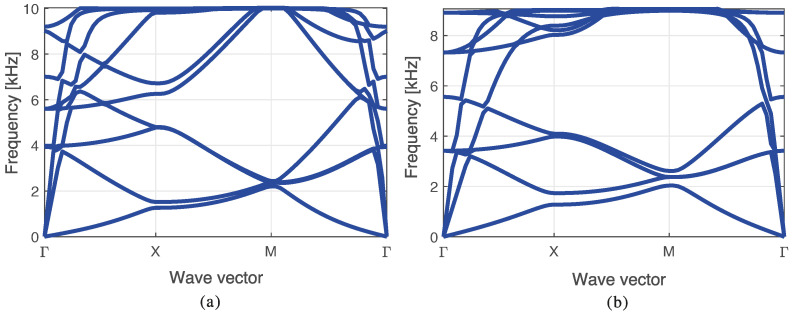
Band diagram of a structure using $n_{c}=2$ and $n_{z}$, rod diameter of $D_{1}$ made entirely of (**a**) aluminum, and (**b**) tungsten.

First, the computation of the band diagrams for non-optimized cores is performed for reference. A topology with $n_{c}=2$, $n_{z}=1$, η=15, and homogeneous cross sections with diameter $D_{1}$ is evaluated for two different materials: aluminum and tungsten, for the first 9 bands. The band diagrams are shown in Figure 12, where the absence of band gaps can be seen. It can also be seen that the frequencies for aluminum are higher than the ones obtained with tungsten, due to its lower mass.

**Figure 13:**
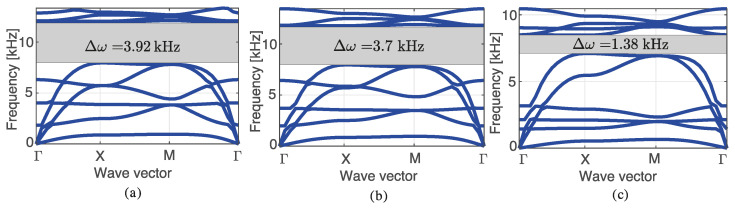
Post-processing effect on the band diagram using $n_{c}=2$, $n_{z}=1$, n=6 with a refinement of η=15, (**a**) no post-processing, (**b**) material post-processing, and (**c**) material and diameter post-processing.

Figure 13 shows the evolution of the band diagram of an optimized structure using $n_{c}=2$, $n_{z}=1$, n=6, and η=15, throughout the post-processing procedure. Figure 13a shows the band diagram obtained without any post-processing for reference. Figure 13b shows the band diagram when post-processing the material (aluminum or tungsten), without changing the geometry. Finally, Figure 13c shows the band diagram obtained when both the material and the geometry are considered in the post-processing procedure. It can be noticed that post-processing the material has little impact in the band gap. Nonetheless, the modification of the geometry has a large impact on the final result. Thus, as discussed in this manuscript, only materials are post-processed since there is no constraint in using diameters in the range $\left[D_{1},D_{2}\right]$.

The best four normalized bandgaps obtained in the previous section are selected for calculating the band diagram and core topology. The bandgaps were selected using the refinement η=15 and a normalized bandgap greater than 0.3. All of the bandgap configurations are presented in Table 4, wherein the normalized, absolute, and mean bandgap frequencies are presented. It can be observed that the objective function formulation, which includes the mean frequency, allows for the generation of proportional bandgaps. Obtaining a bandgap of 1 kHz tuned to 10 kHz is not the same as obtaining a bandgap located at 3 kHz.

**Figure 14:**
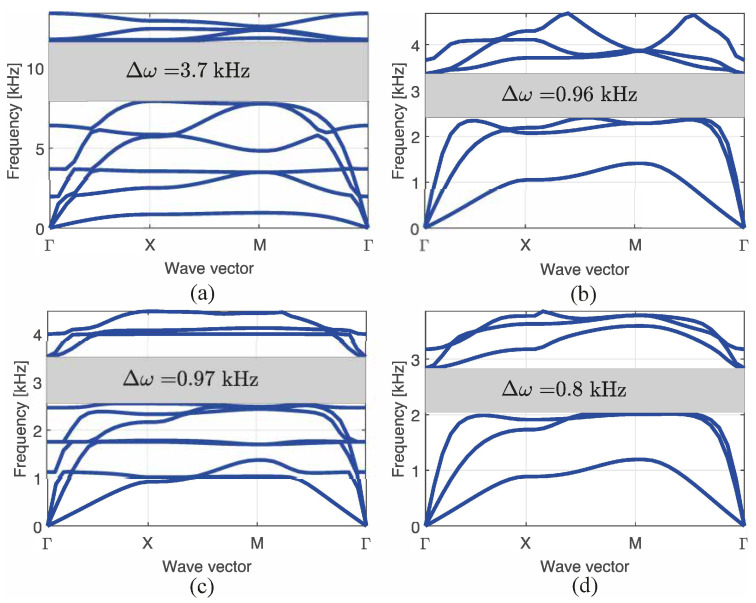
Bandgap of the structure for the best 4 study cases, in which (**a**) $n_{c}=2$, $n_{z}=1$, n=6 (**b**) $n_{c}=4$, $n_{z}=2$, n=3 (**c**) $n_{c}=4$, $n_{z}=2$, n=7, and (**d**) $n_{c}=4$, $n_{z}=3$, n=3.

The band diagrams are shown in Figure 14, in which the frequencies are presented as a function of the wave vector. It is evident that when the mass increases, the mean bandgap frequency decreases. To clearly show the bandgap obtained in the sandwich configuration with $n_{c}=4$ and $n_{z}=3$, only six bands were plotted. In all the other cases, the number of bands was 10. The aforementioned bandgap is the smallest of all obtained bandgaps because it is tuned in a lower frequency; however, its mean bandgap frequency is similar to the other bandgaps.

It should be noted that both bandgaps shown in Figure 14b,c are from the same configuration of $n_{c}=4$ and $n_{z}=2$ and are tuned to similar frequencies, even though the bandgap for the optimized *n* is different (n=3 and 7). These bandgaps differ owing to a slight increase in the mean bandgap frequency at 154 Hz, while the widths of the bandgaps are similar.

The wider bandgap presented in Figure 14a was achieved for the simplest structure ($n_{c}=2$ and $n_{z}=1$). This indicates that the manufacturing of a complex configuration to create a bandgap may not be necessary. Such a structure will only be needed if more mass must be added to the total structure. However, if a lower mean bandgap frequency is needed, the structure may be scaled to a proper size. Therefore, in the aforementioned case, it is not justified to use complicated structures to obtain a bandgap at lower mean frequencies. A careful study of scalability should be conducted to determine how such changes affect the bandgap properties.

**Figure 15:**
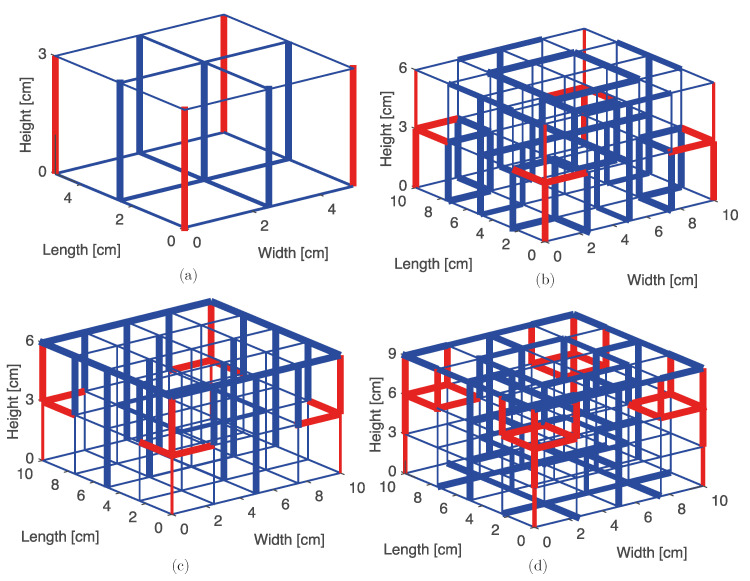
Structure topology for the best 4 study cases in which (**a**) $n_{c}=2$, $n_{z}=1$, n=6; (**b**) $n_{c}=4$, $n_{z}=2$, n=3; (**c**) $n_{c}=4$, $n_{z}=2$, n=7; and (**d**) $n_{c}=4$, $n_{z}=3$, n=3. Red and blue indicate tungsten and aluminum, respectively.

Figure 15 shows the structure topology of the best four study cases noted in the previous section. The material is indicated by using red for tungsten and blue for aluminum. The width of each bar is proportionally represented in the figure.

All of the structures show a reinforcement on their corners, which use tungsten for those elements. A similar behavior was reported in previous literature for 2D structures [13,25,26,32].

## 6. Conclusions

The design of sandwich structures with consideration of vibration reduction was examined in this study. In this regard, the core was optimized using phononic materials to obtain a wide phononic bandgap. Two design variables, namely the area and material properties, were selected for optimization. Different configurations were investigated to study how the number of cells affects the resulting bandgaps, as well as to identify which configuration provides the best results.

The use of a normalized objective function in this problem allows the bandgap to appear at lower frequencies, because the absolute width of the bandgap is not crucial the location at which the bandgap is tuned is more important. The use of simplified structures with a low number of cells is desirable because a simplified manufacturing process may be used. If bandgaps with lower mean frequencies are required, the structure may need to be scaled. However, complex cores may be also required when the structure length is scaled rapidly.

For further investigations, a study of these structure’s scalability is needed in order to consider the range of frequencies in which the bandgaps may be tuned. Moreover, in this study, only two materials with high contrast properties were used; research focused on improving bandgap properties in designs with two materials could be promising in future applications of these structures. This problems does not include structural performance in the formulation, so a multi-objective optimization including the strength, weight, thermal conductivity, among others aspects is a natural continuation of this work. 

## Figures and Tables

**Table 1:** Material, geometry, and parameter selections.

Name	Symbol	Value
Aluminum density	$\rho_{1}$	2700 kg/m^3^
Tungsten density	$\rho_{2}$	19,300 kg/m^3^
Aluminum elastic modulus	$E_{1}$	70 GPa
Tungsten elastic modulus	$E_{2}$	411 GPa
Aluminum Poisson’s ratio	$\nu_{1}$	0.28
Tungsten Poisson’s ratio	$\nu_{2}$	0.33
Lower cross-section diameter	$D_{1}$	2 mm
Upper cross-section diameter	$D_{2}$	8 mm
Cell length	$L_{c}$	2.5 cm
Distance between lattice	$L_{z}$	3.0 cm
P-norm value	*P*	30
Number of eigenvalues considered in the P-norm approximation	*m*	15
Number of points in IBZ	PIBZ	30
Refinement parameter	η	3

**Table 2:** Best normalized bandgaps obtained using the P-norm and post-processing design variables with different refinements.

Number of Cells	Band Maximized *n*	P-Norm	Post-Processed				
			η=3	η=6	η=9	η=12	η=15
$n_{c}=2$, $n_{z}=1$	5	0.325	0.065	0.186	0.284	0.300	0.284
	6	0.339	0.514	0.412	0.391	0.382	0.378
	8	0.355	0.484	0.335	0.303	0.285	0.273
$n_{c}=2$, $n_{z}=2$	3	0.078	0.235	0.188	0.143	0.113	0.094
	4	0.143	0.234	0.206	0.148	0.115	0.100
	8	0.460	0.154	0.061	0.042	0.031	0.021
$n_{c}=4$, $n_{z}=1$	4	0.016	−0.005	−0.059	−0.075	−0.095	−0.109
$n_{c}=4$, $n_{z}=2$	3	0.298	0.362	0.352	0.342	0.336	0.333
	4	0.292	0.349	0.331	0.310	0.297	0.290
	7	0.303	0.348	0.338	0.325	0.320	0.319

**Table 3:** Normalized bandgap for $n_{z}=3$ and n=3 obtained using the P-norm and post-processing design variables with different refinements.

Number of Cells	P-Norm	Post-Processed				
		η=3	η=6	η=9	η=12	η=15
$n_{c}=2$	0.077	0.049	−0.034	−0.116	−0.179	−0.217
$n_{c}=4$	0.309	0.371	0.358	0.342	0.333	0.329

**Table 4:** Absolute and mean bandgaps for all cases with a normalized bandgap greater than 0.3.

Number of Cells	Band-Maximized *n*	Normalized Bandgap	Mean Bandgap Frequency kHz	Absolute Bandgap kHz
$n_{c}=2$, $n_{z}=1$	6	0.378	9.794	3.703
$n_{c}=4$, $n_{z}=2$	3	0.332	2.887	0.961
$n_{c}=4$, $n_{z}=2$	7	0.319	3.041	0.972
$n_{c}=4$, $n_{z}=3$	3	0.329	2.433	0.8

## Data Availability

Not applicable.
